# Long-term exposure to traffic noise and the incidence of hypertension: a systematic review and meta-analysis of prospective cohort studies

**DOI:** 10.3389/fcvm.2026.1834139

**Published:** 2026-05-28

**Authors:** Yumo Xia, Ze Yang, Hongmo Xia, Xiaoyu Ma, Chongchai Li, Qiutong Guo, Jingjing Lu, Mingxue Zhang, Yongliang Xia

**Affiliations:** 1 The First Clinical College, Liaoning University of Traditional Chinese Medicine, Shenyang, Liaoning, China; 2 The First School of Clinical Medicine, Zhejiang Chinese Medical University, Hangzhou, Zhejiang, China; 3 School of Acupuncture-Moxibustion and Tuina, Beijing University of Chinese Medicine Dongfang College, Huanghua, Cangzhou, Hebei, China; 4 Department of Integrated Traditional Chinese and Western Medicine, Liaoning Cancer Hospital, Shenyang, Liaoning, China; 5 Department of Cardiology (No.2 Ward), Liaoning Hospital of TCM, Shenyang, Liaoning, China; 6 TCM Internal Medicine, The First Affiliated Hospital of Zhejiang Chinese Medical University (Zhejiang Provincial Hospital of Chinese Medicine), Hangzhou, Zhejiang, China

**Keywords:** cohort studies, hypertension, incidence, meta-analysis, traffic noise

## Abstract

**Background:**

Hypertension is the major global preventable risk factor in cardiovascular diseases. While traffic noise is a highly disruptive environmental stressor in contemporary cities, its longitudinal causal association with hypertension remains debatable in past research. This study aims to conduct a systematic review and meta-analysis of high-quality cohort studies to evaluate the correlation between long-term traffic noise exposure and the risk of hypertension in adults.

**Methods:**

PubMed, Embase, and Web of Science databases were systematically searched to identify relevant published literature. Prospective or retrospective cohort studies examining the relationship between long term exposure to traffic noise with the occurrence of hypertension in adults were incorporated. The quality of the research studies was evaluated based on the Newcastle-Ottawa Scale methodologically. The association was estimated by data pooling with fixed-or random-effect models and estimated by calculating the HR and its 95%CI.

**Results:**

11 cohort studies with 1,353,481 participants were included into the present research. The results indicated that in cohorts analyzing traffic noise as a binary variable, higher exposure was significantly associated with an increased risk of hypertension (HR = 1.07, 95% CI: 1.01–1.13, *P* = 0.03). This finding is highly consistent with the meta-analysis results where traffic noise was treated as a continuous variable (HR = 1.03, 95% CI: 1.02–1.04, *P* < 0.00001). While heterogeneity was low in the main analysis, moderate to high heterogeneity was present in specific subgroups, such as night noise, follow-up time, and aircraft noise. Subgroup analyses revealed that the pathogenic effect of traffic noise was robust across different spatial and temporal noise sources, as well as follow-up time, and completely independent of traditional confounding factors such as air pollution.

**Conclusion:**

Long-term exposure to traffic noise is significantly associated with an increased risk of hypertension in the adult population. Incorporating environmental noise assessment and mitigation measures into public health strategies can contribute to the comprehensive management of hypertension in the population.

**Systematic Review Registration:**

https://www.crd.york.ac.uk/PROSPERO/view/CRD420251270952.

## 1 Introduction

Hypertension is the leading preventable risk factor for cardiovascular diseases (such as coronary heart disease and stroke) and premature mortality worldwide (1). Clinically and epidemiologically, the core pathophysiological characteristics of hypertension are hemodynamic abnormalities primarily characterized by progressive atherosclerosis and endothelial dysfunction (2). Before patients exhibit obvious clinical symptoms, persistently elevated blood pressure often has already caused irreversible, latent structural damage to key target organs such as the heart, brain, and kidneys (3). According to the latest pooled analysis by the NCD Risk Factors Collaboration, based on data from over 100 million people worldwide, the number of people aged 30–79 with hypertension doubled between 1990 and 2019, surging to a staggering 1.28 billion (4). More alarmingly, the vast majority of people with hypertension worldwide do not receive effective treatment or achieve adequate control; in 2019, blood pressure control rates were only 18% for men and 23% for women (4). The rising prevalence of hypertension places a heavy economic and healthcare burden on public health systems (5). Although traditional physiological and lifestyle factors—such as genetic susceptibility, poor dietary habits, obesity, and physical inactivity—are widely recognized as classic contributors to hypertension, a growing body of epidemiological evidence suggests that exposure to environmental risk factors also plays a significant role in the onset and progression of cardiovascular disease (6).

Among numerous environmental risk factors, traffic noise (primarily comprising road traffic and aviation noise) has become one of the most prevalent and destructive environmental stressors in modern cities (7). As the global urbanization and industrialization stepped up, the transport systems have grown exponentially with traffic noise turning into omnipresent form of physical pollution. Various noise sources of traffic noise are known to have different acoustic properties: road traffic noise is generally a background noise with low frequencies, and aviation or railway noise is often a burst noise with high levels of sound peaks of high decibel levels (8). These inevitable environmental sounds are able to reach the auditory cortex and continuously be perceived and decoded by limbic system of the brain even when individuals are in the unconscious state like during the nighttime sleep (9). At the pathophysiological level, traffic noise primarily causes sustained cardiovascular damage through “non-auditory pathways” (7). Continuous exposure to environmental noise overstimulates the brain's limbic system and sympathetic nervous system even during unconscious states, leading to chronic stress in the hypothalamic-pituitary-adrenal axis. This abnormal neuroendocrine response triggers systemic oxidative stress and vascular endothelial dysfunction, ultimately leading to irreversible hypertension through increased peripheral vascular resistance (10). However, although these mechanisms have been supported by experimental studies, the longitudinal epidemiological impact of traffic noise on the development of hypertension in the real world remains to be clarified (11).

Over the last few years several observational studies have been conducted to determine the relationship between exposure to long term traffic noise and the development of hypertension. Nonetheless, the currently available epidemiological data are not entirely consistent. Some cohort studies have shown that noise exposure is a longitudinal risk factor for hypertension (12–15),although some studies have failed to observe a significant association (16–20). This heterogeneity can be explained by different methodological variations: different researches may define noise sources (road traffic vs. aircraft) and exposure duration (L_den_ vs. L_night_) diversely, more importantly they may differ greatly in the approaches taken by both research to control possible confounding factors. Traffic noise can be located in close proximity with air pollution (including PM2.5 and NO2), and unhealthy lifestyle choices (including smoking, drinking, and high-salt diets) and physiological predisposition (including BMI) can all be closely coupled with cardiovascular risk (21).The failure to adequately account for the effects of these environmental and individual confounding factors in statistical models may be the core reason for the controversial results in previous studies.

This study aims to conduct a comprehensive systematic review and meta-analysis based on the latest high-quality prospective and retrospective cohort studies to clarify the longitudinal causal relationship between long-term exposure to traffic noise and the incidence of hypertension. The goal is to provide more robust and precise evidence-based medical evidence for urban traffic noise control and environmental prevention strategies for hypertension.

## 2 Methods

This study strictly adhered to the guidelines of the “Preferred Reporting Items for Systematic Reviews and Meta-Analyses” (PRISMA) and the standards of the Cochrane Handbook of Systematic Reviews of Interventions.

### 2.1 Literature search

We conducted a comprehensive computerized search of the three major electronic databases—PubMed, Embase, and Web of Science—covering the period from the inception of each database through April 2026. The search strategy combined controlled vocabulary (MeSH/Emtree) with free-text terms to minimize the risk of omitting relevant literature. The specific search term combinations were structured as follows: (1) Exposure factor (traffic noise): The subject term “Noise, Transportation” was combined with free-text terms “Noises, Transportation” OR “Transportation Noise” OR “Transportation Noises.” To further enhance the sensitivity of the search for specific noise sources, we added the following free-text terms restricted to the title/ abstract fields, including “Traffic Noise” OR “Road Noise” OR “Aircraft Noise” OR “Airplane Noise” OR “Aviation Noise”; (2) Outcome measures (hypertension): The subject term “Hypertension” was combined with free-text terms “Blood Pressure, High” OR “Blood Pressures, High” OR “High Blood Pressure” OR “High Blood Pressures.” Boolean logical operators “AND” were used to link the search terms for exposure factors and outcome measures. Additionally, to ensure the comprehensiveness of the search, we manually traced the reference lists of relevant cohort studies and previous review articles to identify other potential studies meeting the inclusion criteria. The complete search strategy is presented in Supplementary Material Table S1.

### 2.2 Inclusion and exclusion criteria

The screening of studies strictly followed the PICOS principles. Studies included in this meta-analysis must meet all of the following criteria: (1) Study population: Adults (≥18 years) with no history of hypertension at baseline; (2) Exposure: Long-term exposure to traffic noise (including road traffic noise or aircraft noise) assessed at baseline, with a clearly defined sound level measurement method (e.g., L_den_, L_night_); (3) Outcome measure: Incidence of hypertension during the follow-up period; (4) Study design: Published prospective or retrospective cohort studies; (5) Data reporting: Reported risk effects (e.g., HR, OR, or RR) adjusted for potential confounders along with their 95%CI, or provided data sufficient to calculate these effects. Studies meeting any of the following criteria were excluded: (1) study types including cross-sectional studies, case-control studies, ecological studies, reviews, editorials, or conference abstracts; (2) studies that did not report the outcome of hypertension incidence or did not measure traffic noise indicators; (3) studies from which complete effect estimates and their variance estimates could not be obtained (e.g., studies reporting only *P*-values without specific effect estimates and 95% CIs).

### 2.3 Data extraction

Literature screening was conducted independently by two researchers (Yumo Xia and Ze Yang). First, duplicate references were removed using EndNote software; subsequently, a preliminary screening was conducted by reviewing titles and abstracts; finally, a full-text review was performed on potentially eligible studies. Data extraction was also conducted independently by the two researchers. The predefined information extracted included: first author's name, year of publication, country of study, follow-up duration, sample size, baseline characteristics of participants (e.g., mean age, proportion of males), noise source type, noise measurement metrics, number of hypertension cases, and confounding variables adjusted in multivariate models. For effect sizes, risk data were extracted separately for noise treated as a continuous variable (per 10 dB increase) and as a dichotomous variable (comparing the highest exposure group with the lowest exposure group). Any disagreements during data extraction were resolved through discussion; if consensus could not be reached, a third senior researcher (Yongliang Xia) was consulted for a final decision.

### 2.4 Assessment of study quality

This study used the Newcastle-Ottawa Scale (NOS) to assess the methodological quality of the included cohort studies. The scale evaluates studies across three dimensions comprising a total of eight items: selection of the study population (4 points), comparability of groups (2 points), and assessment of outcomes (3 points).

In the study population selection dimension, we paid particular attention to the representativeness of the exposed cohort, specifically whether the participants were drawn from the general community population. In accordance with consensus from previous studies, studies with a total score of 1–3 were classified as having a “high risk of bias” (i.e., low-quality studies); those with a total score of 4–6 were classified as having a “moderate risk of bias” (i.e., moderate-quality studies); and those with a total score of 7–9 were classified as having a “low risk of bias” (i.e., high-quality studies) (22).

The quality assessment process was conducted independently by two researchers(Yumo Xia and Ze Yang), who reached consensus on the criteria for each NOS item prior to evaluation. Any disagreements during the evaluation process were resolved through joint discussion; if consensus could not be reached after discussion, a third senior researcher(Yongliang Xia) was consulted for a final decision. The specific scores for each study on the NOS scale are detailed in Table 2.

### 2.5 Statistical analysis

This study included only prospective or retrospective cohort studies, and that the original literature primarily reported HR and RR for assessing longitudinal incidence risk. In longitudinal epidemiological follow-up studies, when the incidence of the target outcome—such as new-onset hypertension—is relatively low during the observation period, the HR and RR are generally considered to be numerically very similar. Therefore, this study used the general inverse-variance method to directly pool these effect estimates. Prior to quantitative synthesis, all effect estimates and their corresponding 95% CIs were transformed to natural logarithmic form to calculate standard errors and ensure normal distribution. Furthermore, we used the pooled HR and its 95%CI to quantify the association between long-term traffic noise exposure and the incidence of hypertension. For cohort studies that assessed traffic noise as a continuous variable, we standardized the risk effects across studies prior to data pooling to represent the change in hypertension risk corresponding to a 10 dB(A) increase in traffic noise exposure. Given the inherent clinical and methodological heterogeneity among the included cohort studies regarding geographic distribution, baseline demographic characteristics, follow-up duration, and noise exposure assessment metrics (e.g., L_den_, L_night_), we used the Cochran *Q*-test and the *I*^2^ statistic to quantitatively assess statistical heterogeneity among the included studies. Model selection was strictly based on the level of heterogeneity: If *I*^2^ < 50%, indicating low heterogeneity among studies, a fixed-effects model was used for the meta-analysis; if *I*^2^ ≥ 50%, indicating significant heterogeneity, a random-effects model was used to obtain more conservative and robust estimates (23). To explore potential sources of heterogeneity and assess the impact of various factors on effect estimates, we predefined the following two major categories of subgroup analyses: (1) Exposure and follow-up time characteristics: exposure period (nighttime noise vs. all-day noise) and follow-up duration (≥5 years vs. <5 years); (2) Environmental and exposure source characteristics: noise source (road traffic vs. aircraft noise) and whether for adjustment for coexisting air pollution; All statistical analyses were performed using Review Manager (version 5.4; Cochrane Collaboration) software. A *P* < 0.05 for two-sided tests was considered statistically significant. In addition, to identify key confounding factors and avoid over-adjustment bias, this study constructed a directed acyclic graph to guide the selection of subgroup analyses and confounding factor adjustment strategies.

## 3 Results

### 3.1 Process and results of the literature screening

**Figure 1:**
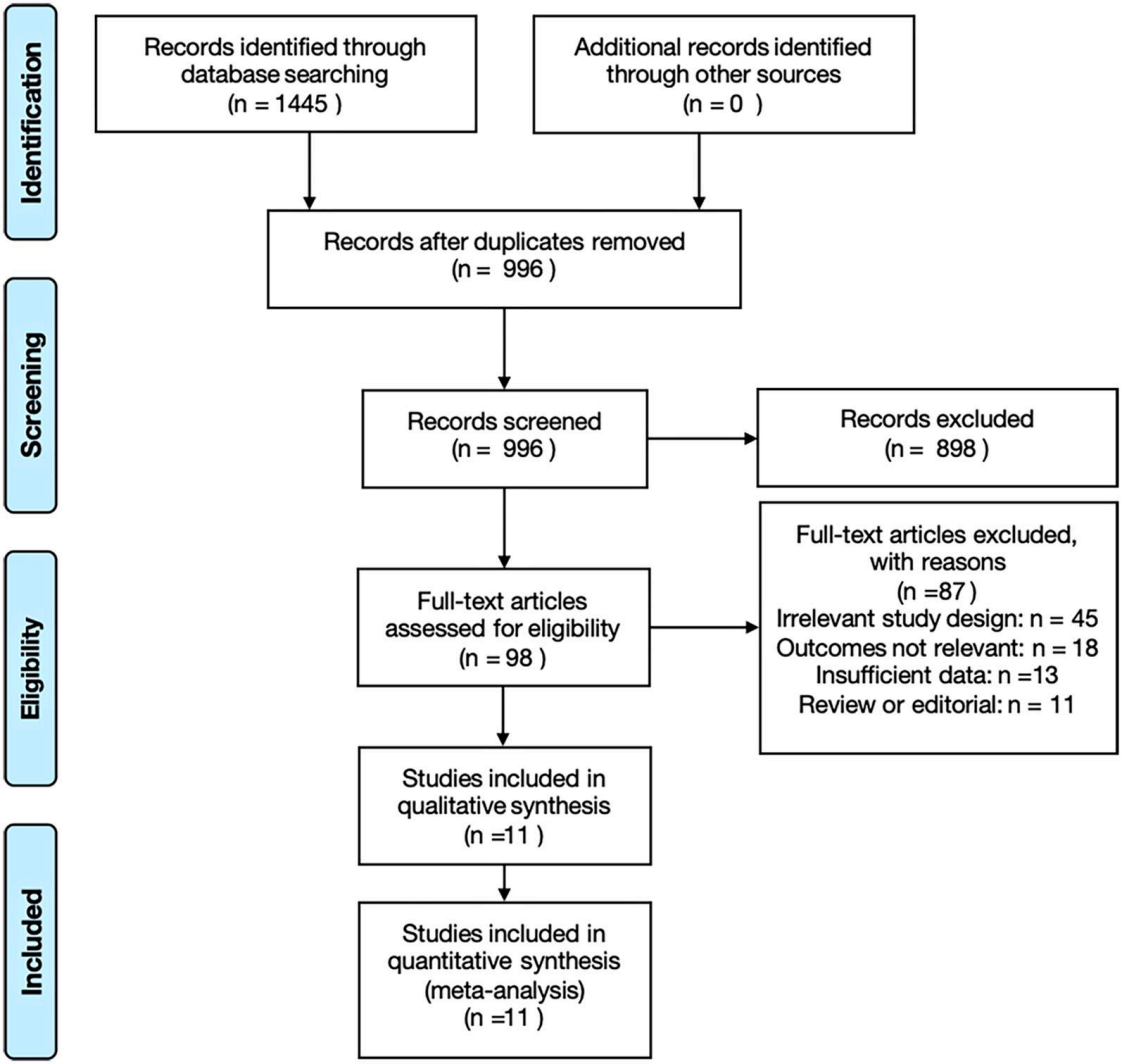
Literature screening of the association between long-term traffic noise exposure and incident hypertension: a PRISMA flow chart.

An initial search of the PubMed, Web of Science, and Embase databases yielded a total of 882 articles (97, 418, and 367, respectively). In addition, a manual screening of the reference lists of relevant reviews and initially included studies did not yield any additional records that met the inclusion criteria. After removing 449 duplicate records using the literature management software EndNote, 996 articles remained for initial screening. The researchers excluded 898 clearly irrelevant articles by reviewing the titles and abstracts. Following a full-text review of the remaining 98 articles, 11 studies were ultimately included in this meta-analysis based on the inclusion and exclusion criteria. The detailed literature screening process is shown in Figure 1.

The main characteristics of the 11 included studies are detailed in Table 1. These studies were published between 2016 and 2025; 10 were prospective cohort studies, and 1 was a retrospective cohort study. The total number of participants across the included studies was 1,353,481, with sample sizes ranging from 430 to 701,174 per study. The follow-up duration was between 4 and 20 years that was long enough to determine the long-term impacts of noise exposure. Geographically, the studies were done in 3 continents: 6 were located in Europe (Sweden, Germany, the United Kingdom, Norway, Greece and France), 4 in North America (United States and Canada) and 1 in Asia (Taipei, China). Concerning population features, the age of the participants were mainly in the middle-aged and old cohort; Regarding gender composition, three studies (Peters et al.; Kim et al.; Nguyen et al.) consisted exclusively of all-female cohorts (0% male proportion).

**Table 1:** Characteristics of the included cohort studies.

Study	Year	Study Design	Country	Follow-up years	Sample size	Mean age (years)	Male (%)	Noise Source	Noise Metric	Proportion of Hypertension (%)	Outcome reported	Type of Effect Estimate	Adjusted variables
Dimakopoulou et al. (17)	2017	PC	Greece	9	420	58.0 ± 9.1	44.3	Road and Aircraft	L_Aeq_ and L_night_	16.90	71	HR	①②③⑥⑦⑧⑬
Fuks et al. (18)	2016	PC	Norway	5.0–8.9 (varying by cohort)	44,917	47.0–71.0 (Mean)	NR	Road Traffic	L_den_	13.40	6,017	RR	①②③⑥⑦⑧⑩⑭
Huang et al. (12)	2023	PC	UK	8.1 (Median)	246,447	55.0 ± 8.1	45.4	Road Traffic	L_den_ and L_night_	8.60	21,140	HR	①②④⑦⑧⑨⑩⑬
Kim et al. (19)	2022	PC	USA	18	162,183	48.2 ± 4.6	0	Aircraft	DNL	37.90	61,445	HR	①③⑨
Kourieh et al. (13)	2022	PC	France	4	853	51.0 (Mean)	45	Aircraft	L_den_	14.80	126	RR	①②③⑥⑧⑪
Nguyen et al. (24)	2023	PC	USA	6 (Median)	18,805	61.3 ± 6.8	0	Aircraft	DNL	44.90	8,441	HR	①④⑦⑧⑨⑩⑪
Peters et al. (25)	2025	PC	USA	20	162,109	59.1 ± 7.1	0	Aircraft	L_night_	37.90	61,445	HR	①③④⑤⑥⑦⑧⑨
Pyko et al. (16)	2018	PC	Sweden	9 (mean)	4,854	53.8 ± 7.7	41	Road Traffic and Aircraft	L_den_	28.70	1,394	HR	No
Shin et al. (14)	2020	RC	Canada	15	701,174	Cohort 1: 51.9 ± 13.0 cohort2: 55.3 ± 14.4	46.8	Road Traffic	L_Aeq,24h_ and L_night_	37.40	262,488	HR	①②③⑤
Tang et al. (15)	2024	PC	Taipei City	4.16	10,773	42.0 (Mean)	48.6	Road Traffic	L_den_	23.40	2,525	HR	①②③④
Voss et al. (20)	2021	PC	Germany	6.5 (mean)	946	56.2 ± 13.1	44	Road Traffic	L_den_	NR	NR	RR	①②⑥⑦⑧⑪⑫

PC, prospective cohort; RC, retrospective cohort; NR: not recorded; L_den,_ A-weighted average 24-hour noise level; L_Aeq,24h_, A-weighted equivalent continuous noise level over 24 h; L_night_, A-weighted equivalent continuous noise level over the night; BMI, body mass index; DNL, day-night average sound level; NO2, nitrogen dioxide; PM2.5, fine particulate matter.

①Age ②Sex ③BMI ④PM2.5 ⑤NO₂ ⑥physical activity ⑦smoking status ⑧alcohol consumption ⑨race ⑩education ⑪occupational status ⑫income ⑬salt intake ⑭family history of hypertension.

According to the evaluation of noise exposure, 7 studies were on road traffic noise, 6 studies were on aircraft noise and some studies combined noise sources on road, aircraft, and other noise sources.

In statistical analyses, the vast majority of studies adjusted for potential confounding factors, including age, sex, BMI, smoking status, and air pollutants (such as PM2.5 and NO2). All included studies were quality-assessed using the NOS, with scores ranging from 7 to 9 (Table 1), indicating that the included literature demonstrated high methodological quality, rigorous study designs, and reliable results.

### 3.2 Quality characteristics, differences, and weakness of the included studies

The 11 cohort studies included in this meta-analysis were of high overall methodological quality, with NOS scores ranging from 7 to 9; however, there were significant differences in study design, population characteristics, and specific limitations.

#### 3.2.1 Major differences among included studies

##### 3.2.1.1 Sample size and geographic distribution

The sample sizes across studies varied greatly, ranging from ultra-large national cohorts with over 200,000 to as many as 700,000 participants, such as Shin et al.; Huang et al.; to small regional studies with fewer than 1,000 participants, such as Dimakopoulou et al.; Kourieh et al.; and Voss et al. The geographic coverage included Europe, North America, and Asia.

##### 3.2.1.2 Study design and noise source assessment

Although 10 of these studies were prospective cohort studies, Shin et al. employed a retrospective cohort design based on administrative health data. Regarding noise sources, the studies focused on road traffic noise, aircraft noise, or a combination of both, and the acoustic indicators used (e.g., L_den_, L_night_) varied, constituting inherent clinical and methodological differences among the studies. As observed in our subsequent meta-analysis, although the primary overall effect exhibited low statistical heterogeneity, these inherent study differences led to moderate to high heterogeneity within certain specific subgroups.

#### 3.2.2 Major limitations of the included studies

Although the overall quality was adequate, the studies revealed various methodological shortcomings (Table 2):

**Table 2:** Details of quality evaluation via the Newcastle–Ottawa Scale.

study	Selection of cohorts			Comparability of cohorts			Outcome of cohorts			Total
	a	b	c	d	e	f	g	h	i	
Pyko et al.	1	1	1	1	1	1	0	1	0	7
tang et al.	1	1	1	1	1	1	1	0	1	8
Voss et al.	1	1	1	1	1	1	0	1	1	8
Huang et al.	1	1	1	1	1	1	1	1	1	9
peters et al.	0	1	1	1	1	1	1	1	1	8
Fuks et al.	1	1	1	1	1	1	0	1	0	7
Kim et al.	0	1	1	1	1	1	1	1	1	8
Nguyen et al.	0	1	1	1	1	1	1	1	1	8
Dimakopoulou et al.	1	1	1	1	1	1	0	1	0	7
Kourieh et al.	1	1	1	1	1	1	1	0	1	8
Shin et al.	1	1	1	1	1	1	1	1	0	8

a. Representativeness of the exposed cohort. b. Selection of the non-exposed cohort. c. Ascertainment of exposure. d. Demonstration that outcome of interest was not present at start of study. e. Comparability of cohorts on the basis of the design or analysis (adjusted for age and gender). f. Comparability of cohorts on the basis of the design or analysis (adjusted for any other factor). g. Assessment of outcome. h. Was follow-up long enough for outcomes to occur (>5 years). i. Adequacy of follow-up of cohorts (>5 years). The scale ranges from one to nine in total, and judge studies above 7 as high-quality cohort studies.

##### 3.2.2.1 Limited population representativeness

As previously mentioned, the studies by Kim et al., Nguyen et al., and Peters et al. were all based on all-female cohorts. Although they demonstrated high internal validity, this limits their external generalizability to men and broader socioeconomic groups.

##### 3.2.2.2 Risk of bias in outcome assessment

According to the outcome assessment item in the NOS score, Dimakopoulou et al., Fuks et al., Pyko et al., and Voss et al. all received lower scores in this category. The reason is that these studies relied on self-reported questionnaires to identify hypertension events rather than rigorous clinical measurements or medical record linkage, which may introduce a degree of classification bias.

##### 3.2.2.3 High dropout rates or inadequate follow-up data

The greatest challenge facing environmental cohort studies spanning several years is participant attrition. Under the NOS follow-up adequacy criterion, the studies by Dimakopoulou, Fuks, Pyko, and Shin all failed to score points due to excessively high dropout rates or a lack of clear reporting on attrition, which may weaken the robustness of their conclusions.

##### 3.2.2.4 Insufficient power in small-sample studies

The studies by Dimakopoulou et al. (*N* = 420) and Kourieh et al. (*N* = 853) had small sample sizes, resulting in wide confidence intervals for their effect sizes and making them more susceptible to random error.

##### 3.2.2.5 Coexisting limitations in exposure measurement

In nearly all included studies, noise exposure levels were estimated using acoustic models based on participants' outdoor residential addresses. This method fails to account for micro-individual factors such as the soundproofing materials of participants' homes, the orientation of bedroom windows, window-opening habits, and occupational noise exposure, thereby inevitably introducing certain measurement errors in exposure assessment.

### 3.3 Meta-analysis results on the association between long-term exposure to traffic noise and hypertension incidence

**Figure 2:**
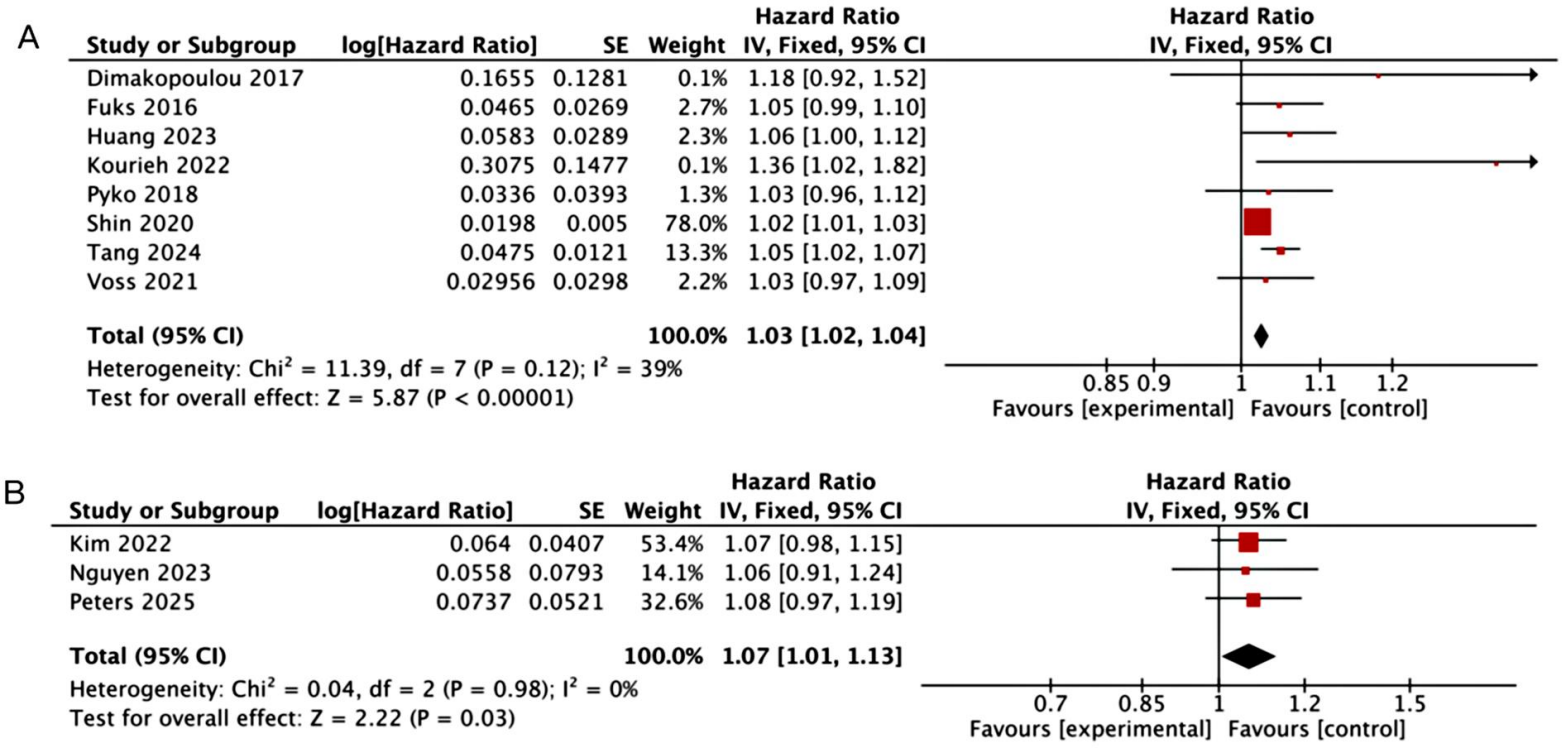
Forest plots of univariate analysis for traffic noise exposure in association with incident hypertension. **(A)** Noise exposure as a continuous variable. **(B)** Noise exposure as a dichotomous variable.

In the overall effect analysis, a fixed-effects model was used for pooling due to low heterogeneity among the included studies (*I*^2^ = 39%). The analysis results showed that in the 8 cohort studies that analyzed noise as a continuous variable, there was a significant positive correlation between traffic noise exposure and the incidence of hypertension (HR = 1.03, 95% CI: 1.02–1.04, *P* < 0.00001; Figure 2A). Similarly, for the three studies that analyzed noise as a dichotomous variable, a fixed-effects model was also adopted due to the absence of heterogeneity within the group (*I*^2^ = 0%); the pooled results also showed that the risk of hypertension increased significantly with rising noise levels (HR = 1.07, 95% CI: 1.01–1.13, *P* = 0.03; Figure 2B).

### 3.4 Results of meta-subgroup analyses on traffic noise and hypertension

#### 3.4.1 Subgroup analyses adjusting for spatiotemporal exposure characteristics and follow-up duration

**Figure 3:**
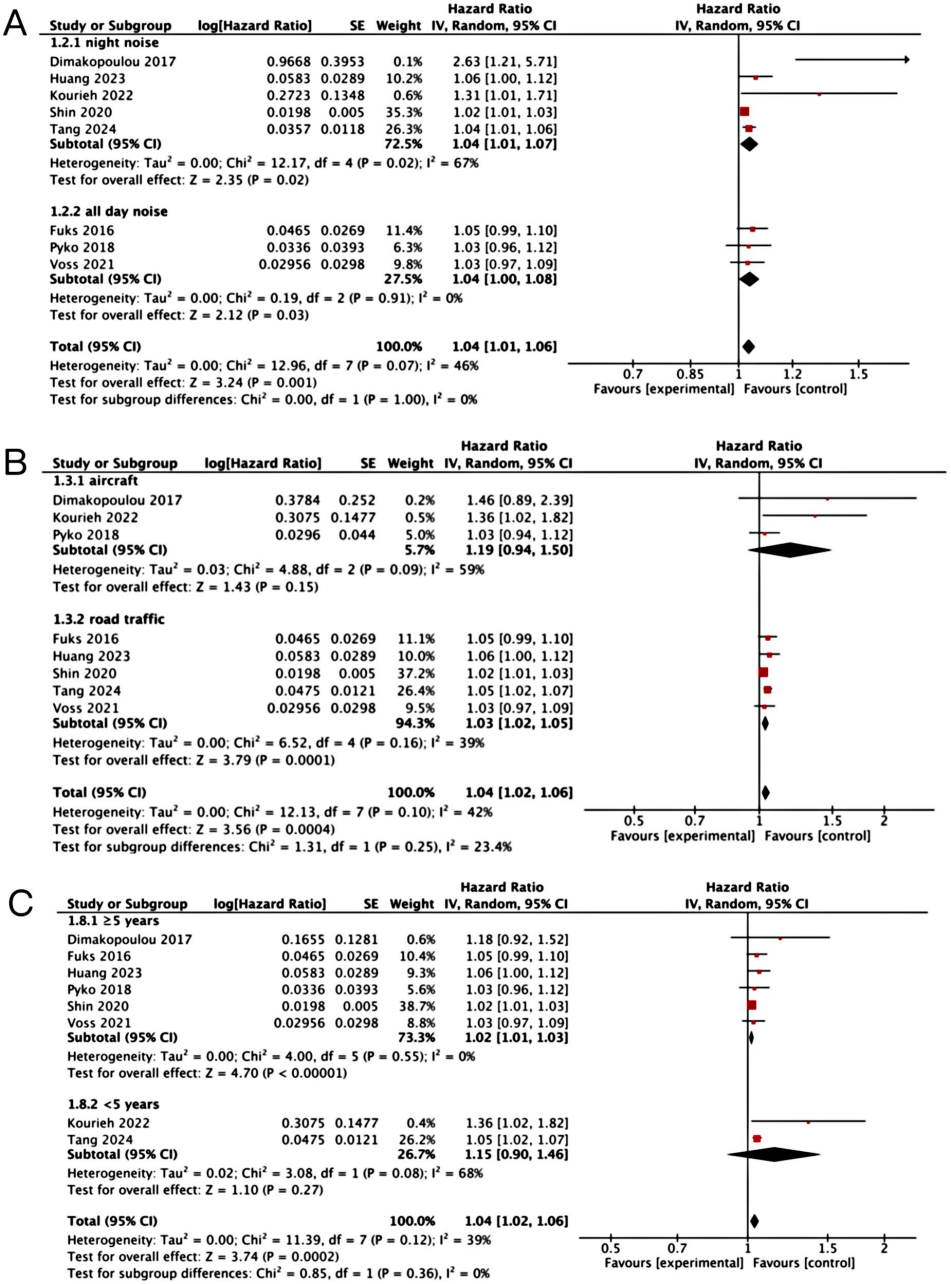
Forest plots of subgroup analyses for the association between traffic noise exposure and incident hypertension, stratified by noise source and follow up duration. **(A)** Subgroup analysis by time source of noise (night, all day). **(B)** Subgroup analysis by source of noise (aircraft, road traffic). **(C)** Subgroup analysis by duration of follow-up (≥5 years vs. <5 years).

Subgroup analysis results based on noise source and time revealed that nighttime noise (HR = 1.04, 95%CI: 1.01–1.07, *P* = 0.02) and all-day noise (HR = 1.04, 95% CI: 1.00–1.08, *P* = 0.03) were both significantly associated with the incidence of hypertension, and there were no significant subgroup differences between these two time periods (*P* = 1.00, Figure 3A).

Subgroup analysis by noise source revealed that road traffic noise significantly increased the risk of hypertension (HR = 1.03, 95% CI: 1.02–1.05, *P* = 0.0001); whereas aviation noise showed a trend toward increased risk but did not reach statistical significance (HR: 1.19, 95% CI: 0.94–1.50, *P* = 0.15). There was no significant difference between the two subgroups (*P* = 0.25, Figure 3B).

Studies with a follow-up duration of ≥5 years showed a significant association between traffic noise and hypertension (HR = 1.02, 95% CI: 1.01–1.03, *I*^2^ = 0%, *P* < 0.00001); while studies with a follow-up duration <5 years also suggested an increased risk, but did not reach statistical significance due to wide confidence intervals (HR = 1.15, 95% CI: 0.90–1.46, *P* = 0.27). No significant difference was observed between the two subgroups (*P* = 0.36, Figure 3C).

When examining objective characteristics related to time and space, given the high heterogeneity within the subgroups for the exposure time period (nighttime noise group, *I*^2^ = 67%), follow-up duration (<5 years group, *I*^2^ = 68%), and aircraft noise group (*I*^2^ = 59%), a random-effects model was uniformly adopted for the analysis in this section.

#### 3.4.2 Subgroup analysis for adjustment of confounding factors

**Figure 4:**
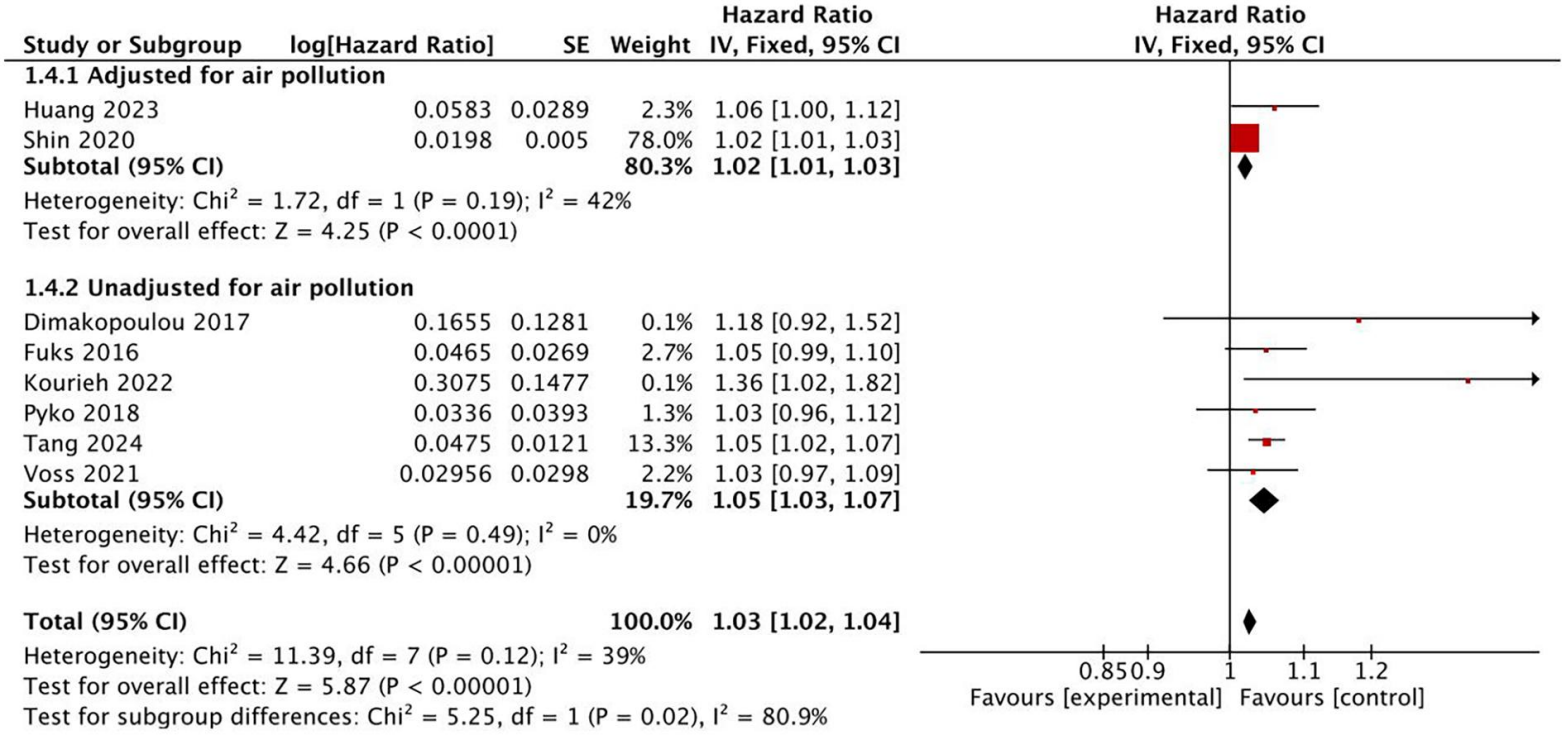
Forest plot of subgroup analysis for the association between traffic noise and incident hypertension, stratified by adjustment for air pollution.

Due to low intra-group heterogeneity (maximum *I*^2^ = 42%, Figure 4), a fixed-effects model was used for this analysis. The results showed that adjusting for air pollution (HR = 1.02, 95% CI: 1.01–1.03, *P* < 0.0001) and studies that did not adjust for this variable (HR = 1.05, 95% CI: 1.03–1.07, *P* < 0.00001) both indicated that traffic noise is an independent risk factor for hypertension, and there was a statistically significant difference (*P* = 0.02).

**Figure 5:**
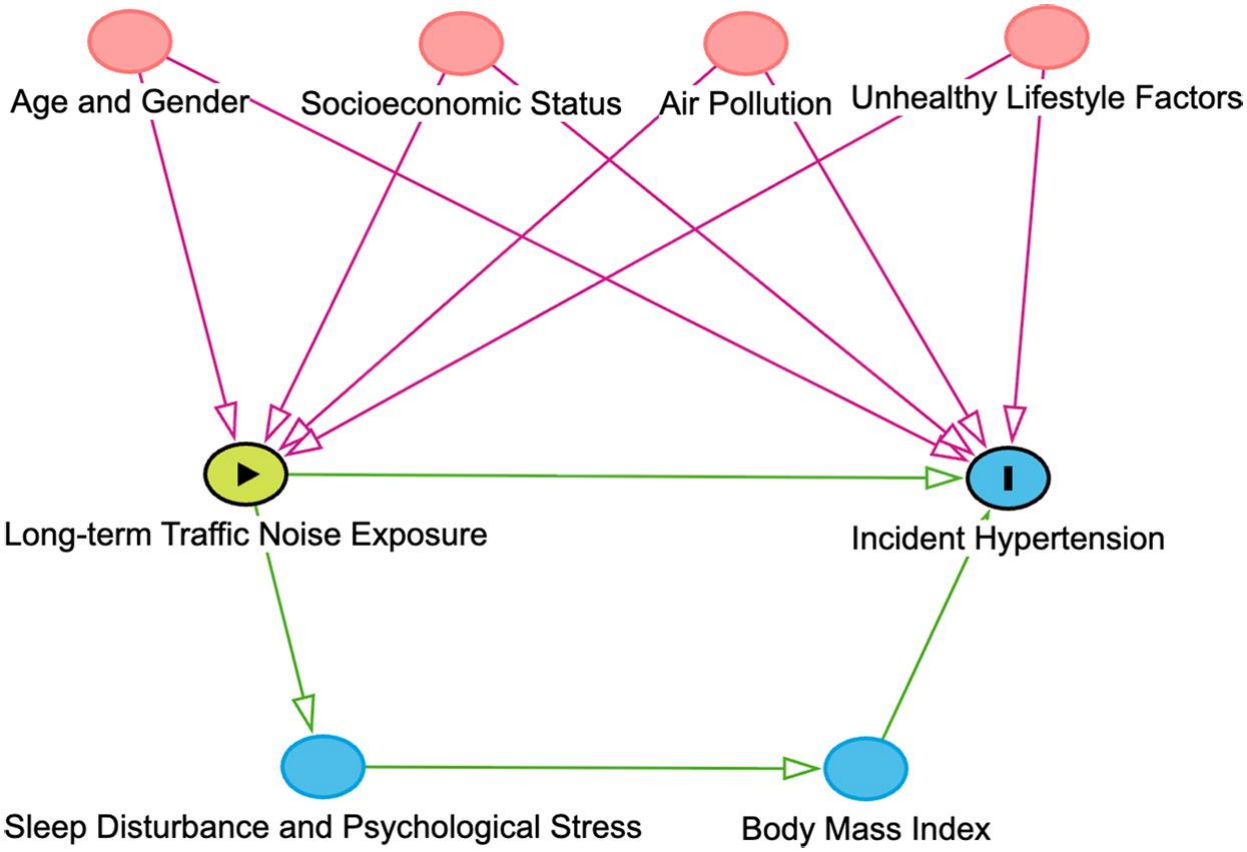
Directed acyclic graph (DAG) illustrating the hypothesized causal pathways and confounding relationships among long-term traffic noise exposure, incident hypertension, and key covariates (age, gender, socioeconomic status, air pollution, unhealthy lifestyle factors, body mass index, sleep disturbance, and psychological stress).

To further enhance the rigor of the statistical analysis, this study introduced Directed Acyclic Graph (Figure 5) to identify key confounding factors and clarify complex pathogenic pathways. During the construction of the causal model, we found that BMI plays an extremely complex role in the association between traffic noise and hypertension. Based on evidence from environmental epidemiology and cardiovascular physiology, long-term exposure to traffic noise—particularly at night—can lead to fragmented sleep and circadian rhythm disruption, which in turn results in suppressed leptin secretion and abnormal fluctuations in cortisol and ghrelin levels. This neuroendocrine dysregulation ultimately contributes to central obesity and metabolic syndrome (26). Consequently, BMI should be regarded as a key mediating variable between noise exposure and the onset of hypertension, following the pathological chain: “traffic noise → sleep/endocrine disruption → obesity → hypertension.” According to epidemiological causal inference theory, forcing adjustment for such an intermediate mediator in the causal pathway within a statistical model can induce significant over-adjustment bias, thereby masking the true epidemiological association of noise exposure (27).

Guided by the aforementioned causal inference framework and rigorous methodological considerations, we formulated the subgroup analysis strategy for this study. First, we explicitly excluded subgroup analyses for BMI and unhealthy lifestyle habits such as smoking and alcohol consumption, as forcibly adjusting or stratifying these mediating variables and complex behavioral covariates within the causal network is highly likely to yield misleading statistical inferences. Second, although socioeconomic status is a key potential confounder, inconsistencies in the definition and adjustment strategies for this variable across the included original studies precluded a pooled analysis of this subgroup. Furthermore, regarding gender—an important demographic characteristic—although this study included three all-female cohorts, all three studies assessed effect sizes based on dichotomous variables; in contrast, the remaining eight studies involving mixed-gender populations reported effect sizes using continuous variables. Since effect estimates derived from binary exposure variables cannot be directly combined or scientifically compared with those from continuous exposure variables, and given the inherent heterogeneity in the structure of the raw data, a gender subgroup analysis could not be performed. Ultimately, while fully balancing clinical significance and data consistency, this study retained and conducted only the air pollution subgroup analysis, aiming to verify whether the association between traffic noise and an increased risk of hypertension remains robust after excluding confounding from related environmental co-exposures.

### 3.5 Sensitivity analysis

To verify the robustness and reliability of the main findings, we conducted a rigorous stepwise sensitivity analysis. Given that the main analysis included two large cohorts—the studies by Shin et al. and Tang et al.—which accounted for an disproportionately high proportion of the total weight, performing this analysis was particularly necessary. Our quantitative assessment indicates that sequentially excluding these high-weight studies did not fundamentally alter the direction or statistical significance of the overall effect size. After excluding the study by Shin et al., the pooled HR was 1.05 (95% CI: 1.03–1.07, *P* < 0.0001), and *I*^2^ decreased to 0%. Similarly, after excluding the study by Tang et al., the pooled HR was 1.02 (95% CI: 1.01–1.03, *P* < 0.00001), with *I*^2^ at 22%. These results confirm that the main effect—that long-term exposure to traffic noise increases the risk of hypertension—remains robust and is not dominated by any single large cohort study. Furthermore, in accordance with best practices in epidemiological methodology and to prevent statistically unstable and potentially misleading interpretations, the stepwise exclusion sensitivity analysis in this study was strictly confined to the main analysis of the overall population and was not conducted within specific subgroups with a limited number of included studies.

### 3.6 Publication bias

According to the Cochrane Handbook for Systematic Reviews of Interventions, when a meta-analysis includes fewer than 10 studies, tests for funnel plot asymmetry lack sufficient statistical power to distinguish between publication bias and chance heterogeneity. When performing the overall effect analysis of this study, the meta-analysis comparing the continuous variables captured 8 studies, whereas the meta-analysis comparing the dichotomous variables captured only 3 studies; none of the two numbers were sufficient to construct a funnel plot. Consequently, to uphold the rigor of the statistics, the study did not consider funnel plots and other comparable quantitative tests (including Egger or Beggs tests) to evaluate the publication bias.

## 4 Discussion

In this systematic review and meta-analysis encompassing 11 high-quality cohort studies, we demonstrate a significant longitudinal association between long-term exposure to traffic noise and an increased risk of hypertension. This robust association remains significant across different noise sources, especially nighttime noise, as well as over longer follow-up periods. The results showed that whether noise was analyzed as a continuous variable (HR = 1.03) or as a binary variable (HR = 1.07), there was a significant positive correlation between traffic noise exposure and the incidence of hypertension. This indicates that as the intensity of environmental noise exposure increases, the risk of hypertension in the population rises accordingly; and that individuals living long-term in areas with high noise pollution face a substantial threat to their cardiovascular health. The primary outcome measure of this study—the incidence of hypertension—exhibited low and manageable heterogeneity (*I*^2^ = 39%), indicating that the overall effect of long-term exposure to traffic noise on increasing the risk of hypertension is highly consistent and reliable across different cohort populations. However, although the main effect was robust, further subgroup analyses revealed moderate to high heterogeneity (*I*^2^ ≥ 50%) in some subgroups, with significant statistical differences observed between them. Therefore, we conducted an in-depth analysis through comprehensive subgroup comparisons and stepwise exclusion sensitivity analyses.

First, the results of the subgroup analysis based on noise source indicate that the subgroup exposed to nighttime noise demonstrated extremely robust statistical significance; the exclusion of any single study did not alter these findings. This highlights the extreme vulnerability of the cardiovascular system to environmental stressors during sleep, a period of physiological recovery. However, this subgroup exhibits high heterogeneity (*I*^2^ = 67%), which may stem from inherent differences in the precision of nocturnal exposure measurements across different cohorts. Therefore, although the overall cardiovascular damage caused by nighttime noise is clear, prospective studies based on more refined individual-level exposure assessments are still needed in the future to further calibrate and validate its exact effect size. Secondly, in the subgroup analysis of noise sources (road traffic vs. aircraft), sensitivity analysis precisely identified the core contributors to heterogeneity. The epidemiological basis for this phenomenon lies in fundamental differences in the physical characteristics of noise exposure across different cohort studies. Road traffic noise typically manifests as continuous, low-frequency background noise present around the clock, whereas aviation noise is characterized by intermittent, high-intensity peaks. As revealed by the study by Pyko et al. (16), intermittent aviation noise peaks may elicit acute autonomic arousal responses more readily than continuous road traffic noise, which explains why the risk effects associated with different noise sources exhibit significant variability. Discussions regarding the duration of follow-up also highlight the cumulative exposure characteristics of traffic noise in disease development. Analysis indicates that in long-term cohort studies with a follow-up duration of ≥5 years, the positive association between traffic noise exposure and the risk of hypertension is extremely robust. Conversely, in the subgroup with a follow-up duration of <5 years, although risk estimates suggested an upward trend, the confidence intervals were wide and did not reach statistical significance. This discrepancy is pathophysiologically plausible, as vascular lesions triggered by environmental noise stress represent a prolonged and progressive chronic process (26). A shorter follow-up window may not be sufficient for such subclinical damage to accumulate and progress into hypertension-related endpoints meeting clinical diagnostic criteria, suggesting that future environmental epidemiological studies should ensure sufficiently long follow-up periods.

Although previous meta-analyses by van Kempen et al. (28). have explored similar topics, their earlier publication date prevented them from incorporating the large, high-quality epidemiological cohort evidence that has emerged in recent years. More critically, the early evaluation had significant limitations in its research framework: on one hand, it included a large number of cross-sectional studies, which cannot establish a clear temporal sequence between exposure and outcome, thus limiting the level of evidence; on the other hand, the exposure source in that study was limited to road traffic noise, failing to comprehensively reflect the broader environmental exposure characteristics such as aviation or mixed traffic noise that modern residents face. These inherent epidemiological limitations directly constituted the fundamental motivation for us to conduct this updated systematic review. To achieve substantial breakthroughs in this field, this study has implemented several core innovations methodologically compared to previous literature. First, we strictly limited the inclusion to high-quality cohort studies with a NOS score of ≥7, a strategy that effectively overcame the common methodological inherent flaws in cross-sectional studies, namely unclear temporal logic and causal inversion. Additionally, we introduced a rigorous directed acyclic graph framework to clarify the boundaries between core baseline confounding factors and pathological mediating variables. This advanced analytical strategy successfully avoided the common “over-adjustment bias” found in previous literature, particularly critical when dealing with metabolic variables such as BMI. Through these strict methodological improvements, this meta-analysis provides more robust and higher-level evidence, further indicating a significant correlation between long-term exposure to traffic noise and increased risk of hypertension. Finally, our findings have extremely urgent public health significance; in the face of the accelerating global urbanization process and a population of 1.28 billion with hypertension, solely relying on correcting individual unhealthy lifestyles is insufficient to comprehensively curb the spread of cardiovascular diseases.

Furthermore, the total number of cohort studies meeting the stringent inclusion criteria is relatively limited; for example, there are only two studies in the subgroup with follow-up times of less than five years, which somewhat limits the statistical power of certain subgroups. Secondly, there are objective differences in the software and algorithms used for calculating traffic noise acoustic models across different countries and regions, which may introduce a degree of exposure measurement error. Additionally, although railway noise is a common and significant source of traffic noise, the included studies did not specifically separate and investigate its independent impact on outcome incidence. The current evidence base is heavily reliant on road and aviation noise, highlighting a key gap that future longitudinal studies need to fill. Moreover, while exploring gender differences in noise-induced hypertension risk has strong clinical relevance, this study was unable to perform gender-based subgroup analyses. This is because the three all-female cohort studies included only reported dichotomous exposure effect values, while the other included studies used continuous variables. This confounding data structure prevented the derivation of robust statistical conclusions for quantitative gender subgroups, thus this study did not conduct gender subgroup analyses. Since part of the merged sample size in this study comes from these three all-female cohorts, and given the potential physiological and endocrine gender differences in environmental stress-induced cardiovascular responses, this may somewhat limit the external generalizability of our overall results to male populations and broader social groups. Future environmental epidemiological research may need to conduct more investigations based on gender-balanced or male-exclusive cohorts to comprehensively elucidate the gender-specific cardiovascular effects of noise exposure. Finally, although most original studies adjusted for core confounding factors, the strategies for adjusting key variables such as socioeconomic status, degree of urbanization, and residential sound insulation conditions were inconsistent across studies, thus it was not possible to completely exclude the influence of these residual confounding factors.

## 5 Conclusion

In summary, based on high-quality longitudinal cohort evidence, this study demonstrates a significant epidemiological association between long-term exposure to traffic noise and an increased risk of hypertension in the adult population. This robust association remained significant even after adjusting for relevant ambient air pollution, and a particularly strong effect was observed during nighttime exposure periods. These findings suggest that environmental traffic noise is an environmental factor that cannot be overlooked in cardiovascular public health. Appropriately incorporating environmental noise exposure assessment and mitigation considerations into broad public health strategies may contribute to a more comprehensive management of hypertension at the population level.

## Data Availability

The original contributions presented in the study are included in the article/Supplementary Material, further inquiries can be directed to the corresponding author.
