# Supplementary material for: Long-term exposure to traffic noise and the incidence of hypertension: a systematic review and meta-analysis of prospective cohort studies
Source: Front Cardiovasc Med. 2026 May 28;13:1834139. doi: 10.3389/fcvm.2026.1834139 (PMC13253683; doi:10.3389/fcvm.2026.1834139)
Supplement: Supplementary file 1 [file Datasheet1.docx]

**Supplementary Table 1**.

Search strategy in PubMed Database

| Number | Search terms |
| --- | --- |
| #1 | "Noise, Transportation"[Mesh] |
| #2 | Noises, Transportation[Title/Abstract] |
| #3 | Transportation Noise[Title/Abstract] |
| #4 | Transportation Noises[Title/Abstract] |
| #5 | Traffic Noise[Title/Abstract] |
| #6 | Road Noise[Title/Abstract] |
| #7 | Aircraft Noise[Title/Abstract] |
| #8 | Airplane Noise[Title/Abstract] |
| #9 | Aviation Noise[Title/Abstract] |
| #10 | #1 OR #2 OR #3 OR #4 OR #5 OR #6 OR #7 OR #8 OR #9 |
| #11 | "Hypertension"[Mesh] |
| #12 | Blood Pressure, High[Title/Abstract] |
| #13 | Blood Pressures, High[Title/Abstract] |
| #14 | High Blood Pressure[Title/Abstract] |
| #15 | High Blood Pressures[Title/Abstract] |
| #16 | #11 OR #12 OR #13 OR #14 OR #15 |
| #17 | #10 AND #16 |

Search strategy in Web of Science Database

| Number | Search terms |
| --- | --- |
| #1 | "Noise, Transportation"[Topic] |
| #2 | Noises, Transportation[Topic] |
| #3 | Transportation Noise[Topic] |
| #4 | Transportation Noises[Topic] |
| #5 | Traffic Noise[Topic] |
| #6 | Road Noise[Topic] |
| #7 | Aircraft Noise[Topic] |
| #8 | Airplane Noise[Topic] |
| #9 | Aviation Noise[Topic] |
| #10 | #1 OR #2 OR #3 OR #4 OR #5 OR #6 OR #7 OR #8 OR #9 |
| #11 | "Hypertension"[Topic] |
| #12 | Blood Pressure, High[Topic] |
| #13 | Blood Pressures, High[Topic] |
| #14 | High Blood Pressure[Topic] |
| #15 | High Blood Pressures[Topic] |
| #16 | #11 OR #12 OR #13 OR #14 OR #15 |
| #17 | #10 AND #16 |

Search strategy in Embase Database

| Number | Search terms |
| --- | --- |
| #1 | 'traffic noise'/exp |
| #2 | 'highway noise' |
| #3 | 'noise, traffic' |
| #4 | 'noise, transportation' |
| #5 | 'road noise' |
| #6 | 'roadway noise' |
| #7 | 'transportation noise' |
| #8 | 'traffic noise' |
| #9 | 'aircraft noise' |
| #10 | 'airplane noise' |
| #11 | 'aviation noise' |
| #12 | #1 OR #2 OR #3 OR #4 OR #5 OR #6 OR #7 OR #8 OR #9 OR#10 OR#11 |
| #13 | 'hypertension'/exp |
| #14 | 'acute hypertension' |
| #15 | 'arterial hypertension' |
| #16 | 'blood pressure, high' |
| #17 | 'cardiovascular hypertension' |
| #18 | 'controlled hypertension' |
| #19 | 'endocrine hypertension' |
| #20 | 'high blood pressure' |
| #21 | 'high renin hypertension' |
| #22 | 'HTN (hypertension)' |
| #23 | 'hypertensive disease' |
| #24 | 'hypertensive effect' |
| #25 | 'hypertensive reaction' |
| #26 | 'hypertensive response' |
| #27 | 'neurogenic hypertension' |
| #28 | 'preexistent hypertension' |
| #29 | 'salt high blood pressure' |
| #30 | 'salt hypertension' |
| #31 | 'secondary hypertension' |
| #32 | 'systemic hypertension' |
| #33 | #13 OR #14 OR #15 OR #16 OR #17 OR #18 OR #19 OR#20 OR #21 OR #22 OR #23 OR #24 OR#25 OR #26 OR #27 OR #28 OR #29 OR #30 OR #31 OR #32 |
| #34 | #12 AND #33 |
